# Targeting Non-Small Cell Lung Cancer Cells by Dual Inhibition of the Insulin Receptor and the Insulin-Like Growth Factor-1 Receptor

**DOI:** 10.1371/journal.pone.0066963

**Published:** 2013-06-24

**Authors:** Emma E. Vincent, Douglas J. E. Elder, Jon Curwen, Elaine Kilgour, Ingeborg Hers, Jeremy M. Tavaré

**Affiliations:** 1 School of Biochemistry, Medical Sciences Building, University of Bristol, Bristol, United Kingdom; 2 CIRA Discovery, AstraZeneca, Alderley Park, Macclesfield, United Kingdom; 3 School of Physiology and Pharmacology, Medical Sciences Building, University of Bristol, Bristol, United Kingdom; Florida International University, United States of America

## Abstract

Phase III trials of the anti-insulin-like growth factor-1 receptor (IGF1R) antibody figitumumab in non-small cell lung cancer (NSCLC) patients have been discontinued owing to lack of survival benefit. We investigated whether inhibition of the highly homologous insulin receptor (IR) in addition to the IGF1R would be more effective than inhibition of the IGF1R alone at preventing the proliferation of NSCLC cells. Signalling through IGF1R and IR in the NSCLC cell lines A549 and Hcc193 was stimulated by a combination of IGF1, IGF2 and insulin. It was inhibited by antibodies that block ligand binding, αIR3 (IGF1R) and IR47-9 (IR), and by the ATP-competitive small molecule tyrosine kinase inhibitors AZ12253801 and NVPAWD742 which inhibit both IGF1R and IR tyrosine kinases. The effect of inhibitors was determined by an anchorage-independent proliferation assay and by analysis of Akt phosphorylation. In Hcc193 cells the reduction in cell proliferation and Akt phosphorylation due to anti-IGF1R antibody was enhanced by antibody-mediated inhibition of the IR whereas in A549 cells, with a relatively low IR:IGF1R expression ratio, it was not. In each cell line proliferation and Akt phosphorylation were more effectively inhibited by AZ12253801 and NVPAWD742 than by combined αIR3 and IR47-9. When the IGF1R alone is inhibited, unencumbered signalling through the IR can contribute to continued NSCLC cell proliferation. We conclude that small molecule inhibitors targeting both the IR and IGF1R more effectively reduce NSCLC cell proliferation in a manner independent of the IR:IGF1R expression ratio, providing a therapeutic rationale for the treatment of this disease.

## Introduction

Lung cancer is the leading cause of cancer death worldwide with Non-Small-Cell Lung Carcinoma (NSCLC) accounting for approximately 80% of all cases. The overall five year survival rate in Europe is 8% [1] and the median survival after diagnosis is 4–5 months if left untreated [2]. Standard chemotherapy in advanced stage NSCLC provides only marginal improvement in overall survival, however EGFR tyrosine kinase inhibitors improve survival in patients carrying activating mutations in the EGFR gene [3]. Other promising therapeutic targets in NSCLC include anaplastic lymphoma kinase (ALK), histone deacetylation (HDAC) and the IGF (insulin-like growth factor) system [4].

The IGF system plays a crucial role in the regulation of energy metabolism and growth [5]. There are two parental receptors in the IGF system that are active in signalling; the IGF1R and the insulin receptor (IR), both of which exist as homodimers comprising two ‘half receptors’. Due to high sequence homology they are also present as hybrid receptors formed by an insulin half receptor and an IGF1 half receptor in cells expressing both receptor genes [6], [7]. The IR and IGF1R are activated by insulin and IGF-1 respectively, however a third ligand, IGF-2, binds both the IGF1R and a splice variant of the IR called IR-A [8]. A third receptor, IGF2R, has no known signal transduction properties and serves as a clearance receptor for IGF-2 [9]. IGF binding proteins (IGFBPs 1–6) also have an important role to play in regulating the concentration of free ligand and the exposure of a ligand to its receptor [10]. In serum, the majority of circulating IGF-1/2 is complexed with IGFBP3. This protects the growth factors from degradation but can also inhibit their binding to receptors [11]. When activated by ligand binding the receptors initiate signal transduction through their tyrosine kinase activity to downstream cascades such as the RAS/RAF/MAPK pathway and the PI3K/Akt pathway. These pathways are responsible for regulating processors such as foetal development, tissue growth and metabolism [12].

As a central regulator of growth and survival, deregulation of the IGF system is common in human cancer (reviewed in [13]. Excess autocrine/paracrine production of IGF-1 and IGF-2 and/or low IGFBP3 levels are associated with an increased cancer risk of several cancers including breast [14], endometrial [15] and bladder [16]. Studies in this area have primarily investigated the role of the IGF1R. Inhibition of the IGF1R using inhibitory antibodies results in a considerable reduction in proliferation of tumour cell lines [17] and it has been found to be overactive in cancers including prostate [18], breast [19], colon [20] and gallbladder carcinoma [21]. The body of evidence is such that it has led to the investigation of IGF1R inhibitors in more than 70 oncological trials [22]. These inhibitors fall in to two classes; monoclonal antibodies targeting the extracellular domain and small molecule ATP-competitive tyrosine kinase inhibitors designed to selectively inhibit the IGF1R over the IR but possessing activity against both receptors.

Concerns that co-inhibition of the IR by small molecule IGF1R kinase inhibitors would have undesirable metabolic consequences have led to IGF1R-selective monoclonal antibodies being favoured for development and use in clinical trials. Nonetheless, it has been known for some time that insulin may stimulate growth of human cancer cell lines [23]–[26] and that 80% of breast cancers overexpress the IR compared with normal breast tissue [27]. In addition, expression of the foetal isoform of the IR, IR-A, is elevated in several human malignancies and, in contrast to signals mediated by IR-B, which have a predominantly metabolic effect, IR-A has a predominantly proliferative effect [28]. Zhang et al have shown that downregulation of the IR inhibited metastasis of breast cancer cells in an athymic mouse model [29], and recent investigations have begun to assess the benefit of dual inhibition of the IGF1R and IR in models of osteosarcoma [30] and in pancreatic neuroendocrine carcinogenesis [31]. In each case the IR contributed to the cell survival effect of the IGF system and enhanced multistage tumour progression in the pancreatic neuroendocrine system. These studies suggest a role for the IR in tumorigenesis.

In NSCLC the involvement of the IGF system is supported by a number of studies. The IGF1R is frequently expressed [32]–[34], and high IGF-1 and low IGFBP3 levels are associated with higher risk and increased severity of lung cancer [35], [36]. IGF1 has mitogenic effects on some NSCLC cell lines [37], [38], and fibroblast-derived IGF2 promotes the growth of NSCLC cells in vivo [39]. Hence in NSCLC it has been reasoned that the IGF1R may be a viable therapeutic target. In a phase-II study of advanced NSCLC, first-line treatment with the fully humanised monoclonal IGF1R antibody figitumumab (CP-751,871) increased the response rate and progression-free survival benefit of paclitaxel and carboplatin [40]. However, recent NSCLC phase III trials testing figitumumab with either chemotherapy or the EGFR inhibitor erlotinib were suspended due to lack of survival benefit [41]. Given the emerging evidence for a role of the IR in other tumour types, it is possible that this lack of clinical benefit of figitumumab in NSCLC was due, at least in part, from unencumbered signalling through the IR.

In the present study we have determined whether signalling through the IR supports NSCLC tumour cell proliferation when the IGF1R is inhibited. We utilised neutralising monoclonal antibodies that selectively bind the IGF1R or the IR in addition to ATP-competitive small molecule inhibitors that simultaneously inhibit both receptors. This approach also enabled a comparison of the efficacies of antibodies and small molecule inhibitors targeting the IGF-signalling axis in inhibiting tumour cell proliferation.

## Materials and Methods

### Materials

AZ12253801 (Astra Zeneca, Macclesfield, Cheshire, UK), NVP-ADW742 (Selleck Chemicals, Houston, TX, USA) and Akti-1/2 (Merck, Darmstadt, Germany) were made in DMSO (final concentration in experiments was 0.5% (v/v)). αIR3 (Merck Chemicals Ltd., Nottingham) and IR47-9 (kindly supplied by Ken Siddle (University of Cambridge, UK)) were prepared in PBS. Insulin (Sigma, Poole, UK), IGF1 (Gro-Pep, Adelaide, Australia) and IGF2 (R&D Systems, Abingdon, UK) were handled according to the manufacturer’s instructions. Antibodies: p-Akt S473 and IGFRβ were purchased from Cell Signalling Technology (Boston, MA, USA), IRβ from Santa Cruz Biotechnology Inc (Santa Cruz, CA, USA) and α-tubulin from Sigma. Donkey HRP-conjugated anti-mouse IgG and anti-rabbit IgG antibodies were from Jackson ImmunoResearch Laboratories (Bar Harbor, ME, USA).

### Cell Culture and use of Growth Factor Combinations

A549 cells were from ATCC (Teddington, UK) and Hcc193 cells [42]–[44] were a gift from Michael Seckl (Imperial College, London). The cell lines were routinely cultured in “growth medium” consisting of DMEM (A549) or RPMI (Hcc193) supplemented with 10% (v/v) foetal bovine serum (FBS, Invitrogen, Paisley, UK), 20000 U/ml penicillin, 7 mM streptomycin and 200 mM glutamine. Cell lines were grown at 37°C in a humidified atmosphere supplemented with 5% (v/v) CO_2_.

For most of the assays described, cells were exposed to a combination of 1 nM insulin, 50 nM IGF1, 50 nM IGF2 (termed ‘3GF’) in order to better represent the tumour microenvironment. Here insulin and IGFs are delivered by the circulation and IGFs are also produced by the stromal and tumour cells themselves to act in a paracrine and autocrine manner. The concentration of IGF1 and IGF2 in 3GF was based on that found in preliminary experiments to enhance cell proliferation in anchorage-independent growth assays whilst taking in to account the increased IGF bioavailability in the tumour microenvironment due to local production and to tumour-secreted proteases causing hydrolysis of IGF binding proteins [5].

To assess the effect of growth factors and inhibitors on Akt phosphorylation and on receptor expression, cells were seeded in 12-well experimental plates to reach 90% confluence after 24 h. Cells were incubated with various inhibitors for 2 h in serum free media before treatment with growth factors for 10 min. Following incubation, protein was extracted from cells as previously described [45].

### Anchorage-independent Growth Assay

From adherent culture cells were trypsinised and passed through an 18G needle to form a single-cell suspension. These cells were suspended in 0.4% (w/v) noble agar in growth medium containing 0.1% (v/v) FBS and 150µ µl of the suspension added per well of a 96-well plate (Greiner Bio-One, A549 cells: 15000 cells/well, Hcc193 cells: 25000 cells/well) over a solidified 40 µl base layer of 0.6% (w/v) agar. Where required, inhibitors and growth factors were added to the cells at the time of suspension in the growth medium agar solution. Suspension cultures were incubated for 5 days at which point 10% by volume of Alamar Blue (Serotec, Kidlington, UK) was added to the wells and the cultures incubated for a further 2–5 h. Metabolically active cells convert Alamar Blue to a fluorescent indicator so that quantification of fluorescence is a measure of the number of living cells. Fluorescence was analysed using a Perkin Elmer, Fusion plate reader with 544 nm excitation and 590 nm emission filters.

### Western Blotting Analysis

NSCLC cell lysates (15 µg protein) were subjected to SDS-PAGE and western blotting as previously described [45]. Briefly, after separation of lysates using 4–12% Bis-Tris gradient gels (Invitrogen Ltd., Paisley, UK), proteins were transferred to polyvinylidene difluoride membranes (Millipore, Hertfordshire, UK). Membranes were incubated with primary antibody (1 µg/ml) overnight before washing and incubating with the appropriate secondary antibodies for 1 h. Immunoblots were visualised using an Enhanced ChemiLuminescence detection system (ECL; Amersham Biosciences). All primary antibodies used here detect one prominent band at the predicted molecular weight of the protein the antibody is raised against. Scanned images of immunoblots are cropped to feature the prominent band.

### Statistical Analysis

Statistical significance was determined by two-tailed unpaired *t* test. p<0.05 was considered statistically significant.

## Results

### AZ12253801 Inhibits Downstream Signalling from the IGF1R and IR with Equal Potency whereas Inhibitory Monoclonal Antibodies (αIR3 and IR47-9) are Specific to their Target Receptor

In this study we used the inhibitory monoclonal antibodies αIR3 and IR47-9, targeted to IGF1R [46] and IR [7] respectively, and the ATP competitive small molecule kinase inhibitor AZ12253801 that has inhibitory activity towards both receptors. Initial experiments were performed to assess the efficacy and specificity of αIR3, IR47-9 and AZ12253801 in the Hcc193 NSCLC cell line. 24 h post seeding, cells were treated with αIR3, IR47-9 (0.5–10 µg/ml) or AZ12253801 (2–100 nM) and then stimulated with growth factor. A low concentration (5 nM) of growth factor was used so the factors activated only their cognate receptor. The phosphorylation of the downstream serine/threonine kinase, Akt, on serine-473 (S473) was used as a measure of insulin- and IGF1-induced signalling activity.

**Figure 1:**
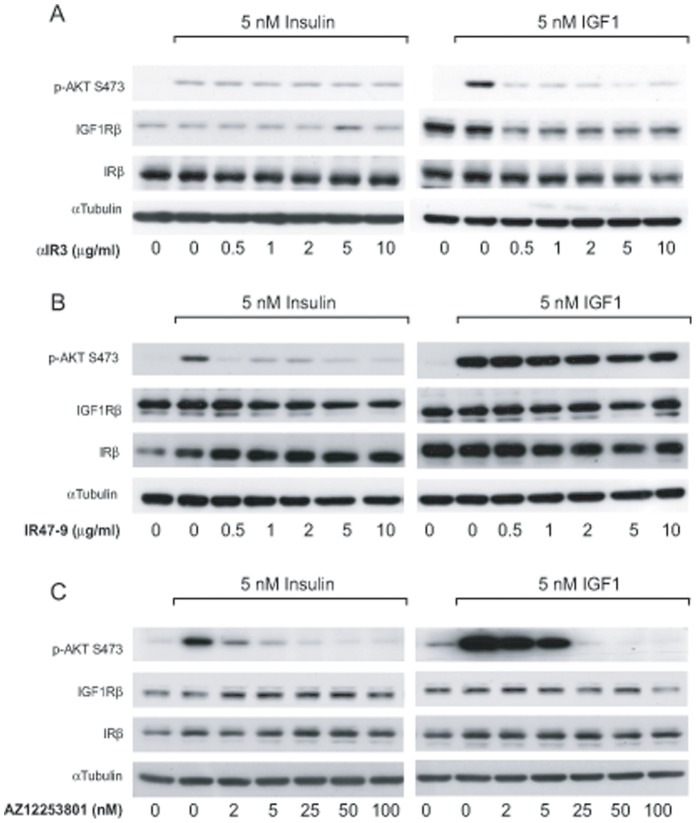
αIR3 and IR47-9 monoclonal antibodies specifically inhibit the IGF1R and the IR respectively whereas AZ12253801 inhibits both receptors in Hcc193 cells. Hcc193 cells were treated either αIR3 (A), IR47-9 (B) or AZ12253801 (C) at the indicated doses for 2 h before 10 min incubation with either 5 nM insulin or 5 nM IGF-1. Phosphorylation of Akt on S473 and expression of IGFRβ and IRβ was determined by western blotting using specific antibodies. An anti-α-tubulin antibody was used as a control for protein loading. All primary antibodies used here detect one prominent band at the predicted molecular weight of the protein the antibody is raised against. Scanned images of immunoblots are cropped to feature this prominent band.

Stimulation with either insulin or IGF-1 caused increased phosphorylation of Akt on S473 (Figure 1A). The IGF1R inhibitory monoclonal antibody, αIR3, inhibited IGF-1, but not insulin-mediated Akt phosphorylation. Conversely, the IR inhibitory monoclonal antibody, IR47-9, inhibited insulin, but not IGF-1, -mediated Akt phosphorylation (Figure 1B). This confirmed that these antibodies inhibit only their respective receptors at the antibody concentrations used in this study. Treatment with αIR3 reduced the expression level of the IGF1R (Figure 1A, right hand panel), which is consistent with previous observations in the MCF7 cell line ([47]; DJEE, EK, JMT unpublished observations).

Pre-treatment of cells with AZ blocked insulin- and IGF1-induced Akt phosphorylation in a dose-dependent manner and with similar potency (Figure 1C). Unlike pre-treatment with inhibitory monoclonal antibodies, AZ12253801 (at 25 nM and above) inhibited Akt phosphorylation to levels below that in untreated cells (Figure 1C). Neither αIR3 or IR47-9 achieved this even at the highest concentration used (10 µg/ml) or when the pre-treatment time (at 2 µg/ml) was extended to 24 h (Figure 1A,B and data not shown). In contrast to αIR3, AZ12253801 did not decrease the expression level of the IGF1R.

### Combined Inhibition of the IGF1R and IR Blocks Cell Growth more Potently than Inhibition of the IGF1R alone in Hcc193 Cells

To investigate a possible contribution of the IR in supporting tumour cell proliferation when the IGF1R is inhibited, Hcc193 NSCLC cells were cultured under anchorage-independent conditions in the presence of IGF1, IGF2 (at concentrations that simultaneously activate both receptors) and insulin (3GF, see Materials and Methods). The effects on proliferation of inhibiting the IGF1R and the IR, alone and in combination, were determined. Maximally effective concentrations of inhibitor were selected based on the data in Figure 1.

**Figure 2:**
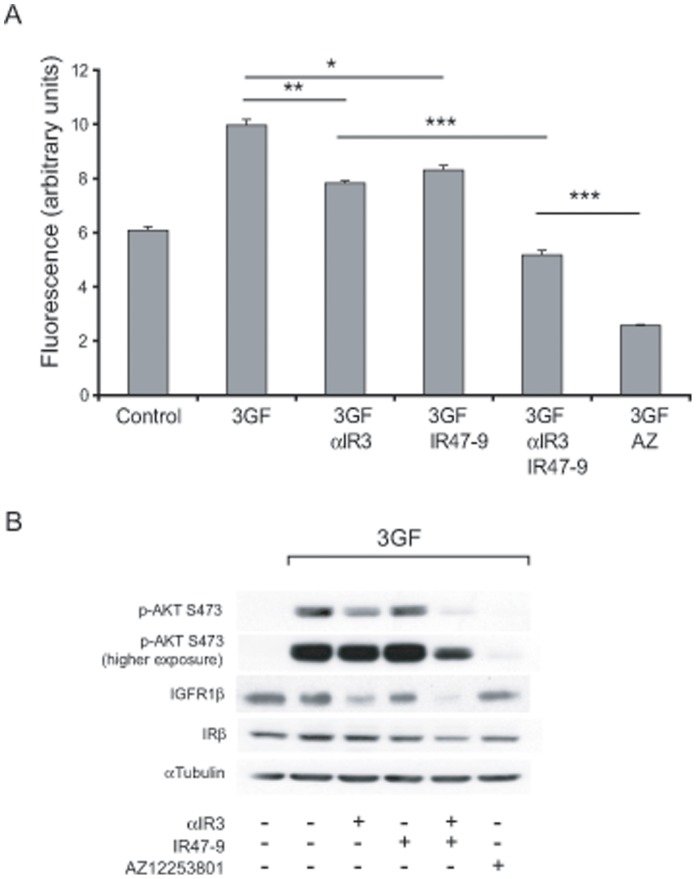
Combined inhibition of the IGF1R and IR more effectively inhibits proliferation of Hcc193 cells than inhibition of IGF1R alone. (A) Hcc193 cells were cultured under anchorage–independent conditions in 0.1% serum in the presence of 3GF (1 nM insulin, 50 nM IGF-1, 50 nM IGF-2) alone and in combination with αIR3 (2 µg/ml), IR47-9 (2 µg/ml), αIR3 (2 µg/ml) and IR47-9 (2 µg/ml), and AZ12253801 (50 nM), as indicated. After 5 days viable cells were estimated by the fluorescence of the metabolic reduction product of Alamar blue. Each bar represents the mean fluorescence of four replicate wells ± standard deviation from a single experiment. The results shown are representative of 3 independent experiments. ***, *p*<1×10^−5^; **, *p*<0.0001; *, *p*<0.01 (valid for all repeats); two-tailed unpaired *t* test. (B) Hcc193 cells were treated with αIR3 (2 µg/ml), IR47-9 (2 µg/ml), αIR3 (2 µg/ml) and IR47-9 (2 µg/ml), and AZ12253801 (50 nM) as indicated for 2 h before 10 min incubation with vehicle or 3GF. Phosphorylation of Akt on S473 and expression of IGFRβ and IRβ was determined by western blotting. An anti-α-tubulin antibody was used as a control for protein loading. All primary antibodies used here detect one prominent band at the predicted molecular weight of the protein the antibody is raised against. Scanned images of immunoblots are cropped to feature this prominent band.

Figure 2A illustrates the proliferation data for Hcc193 cells in anchorage-independent culture. 3GF potentiated the proliferation of Hcc193 cells by 1.6-fold compared to that observed with vehicle alone. Inhibition of the IGF1R by αIR3 or the IR by IR47-9 reduced the proliferation observed in the presence of 3GF (3GF proliferation) by 22% (*p*<0.0001) and 17% (*p*<0.01) respectively. Blocking both the IGF1R and IR with αIR3 and IR47-9 resulted in a 48% reduction in 3GF proliferation, a significantly greater effect than obtained by blockade of either receptor alone (*p*<1×10^−5^ in each case). The finding that IR47-9 blocked proliferation induced by 3GF either alone or in combination with αIR3 suggests that the insulin receptor is directly capable of promoting the proliferation of Hcc193 cells.

Simultaneous blockade of the IGF1R and the IR using AZ12253801 resulted in a greater inhibition of proliferation (75%) than obtained with αIR3 and IR47-9 in combination (*p*<1×10^−5^) (Figure 2A). This may be explained by the increased effectiveness of AZ12253801 in inhibiting 3GF-induced Akt phosphorylation (Figure 2B). Furthermore, the combined antibodies were more effective at inhibiting Akt phosphorylation than was each antibody alone (Figure 2B). Thus, the effect of the inhibitors on 3GF-induced Akt phosphorylation, when added alone or in combination, parallel their effect on 3GF-induced proliferation.

### The IGFR to IR Expression Ratio may Predict the Dependence of Cells on the IR for Survival

**Figure 3:**
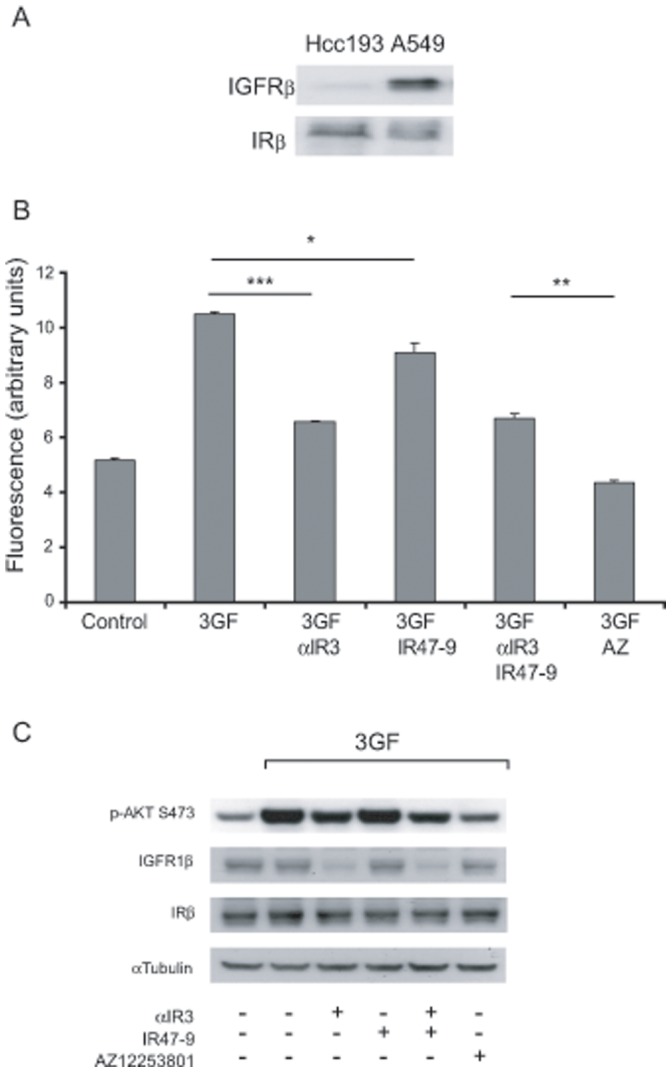
Inhibition of the IGF1R and IR in combination does not inhibit growth of A549 cells more effectively than inhibition of IGF1R alone. (A) Comparison of expression of IGF1Rβ and IRβ in Hcc193 and A549 cell lines was determined by western blotting using 15 µg protein extracted from each cell line. (B) A549 cells were cultured under anchorage–independent conditions in 0.1% serum in the presence of 3GF alone and in combination with αIR3 (2 µg/ml), IR47-9 (2 µg/ml), αIR3 (2 µg/ml) and IR47-9 (2 µg/ml), and AZ12253801 (50 nM), as indicated. After 5 days viable cells were estimated by the fluorescence of the metabolic reduction product of Alamar blue. Each bar represents the mean fluorescence of four replicate wells ± standard deviation from a single experiment. The results shown are representative of 3 independent experiments. ***, *p*<1×10^−6^; **, *p*<0.0001; *, *p*<0.05 (valid for all repeats); two-tailed unpaired *t* test. (C) A549 cells were treated with αIR3 (2 µg/ml), IR47-9 (2 µg/ml), αIR3 (2 µg/ml) and IR47-9 (2 µg/ml), and AZ12253801 (50 nM) as indicated for 2 h before 10 min incubation with vehicle or 3GF. Phosphorylation of Akt on S473 and expression of IGFRβ and IRβ was determined by western blotting. An anti-α-tubulin antibody was used as a control for protein loading. Scanned images of immunoblots are cropped to feature the prominent band.

We next examined whether the relative expression of IGF1R and IR receptors affected the ability of IR47-9 to inhibit tumour cell proliferation or the comparative efficacy of AZ12253801 and the combined neutralising antibodies. To this end another NSCLC line, A549 was used as it expresses approximately equal numbers of IR as Hcc193 cells but significantly more IGF1R, as determined by western blotting equal amounts of lysate (Figure 3A). Densitometric analysis indicated that Hcc193 cells have an IR:IGF1R expression ratio ∼15-fold that of A549 cells.

A549 cells were cultured under the conditions described for Hcc193 (Figure 2). 3GF potentiated the proliferation of A549 cells by 2-fold over that observed for the vehicle control (Figure 3B). Inhibition of the IGF1R by αIR3 reduced 3GF-stimulated proliferation by 40% (*p*<1×10^−6^) approximately 2-fold the reduction seen in Hcc193 cells. In contrast, inhibition of the IR by IR47-9 had a modest effect reducing 3GF proliferation by 10% (*p*<0.05) and, unlike with Hcc193 cells, the presence of IR47-9 failed to enhance the effect of αIR3 on 3GF proliferation (Figure 3B). Thus in A549 cells the IR is much less effective at supporting anchorage-independent proliferation than is observed with Hcc193 cells. Nevertheless, as observed with the Hcc193 cells, the degree of inhibition of A549 cell proliferation by AZ12253801 was significantly greater than that observed in the presence of the antibody combination (p<0.0001; Figure 3B).

The effect of the inhibitors in the A549 anchorage-independent assay was again consistent with their effect on Akt phosphorylation (Figure 3C); αIR3 inhibited 3GF-induced Akt phosphorylation, no additional effect was observed when IR47-9 was combined with αIR3, and AZ12253801 was more effective than αIR3 (or combined αIR3 and IR47-9). αIR3 caused a reduction in expression of IGF1Rβ but not of IRβ in A549 cells (Figure 3C), as previously observed in Hcc193 cells (Figure 1 and 2).

### Effective Inhibition of Cell Proliferation by a Small Molecule IR/IGF1R Inhibitor Structurally Distinct to AZ12253801

**Figure 4:**
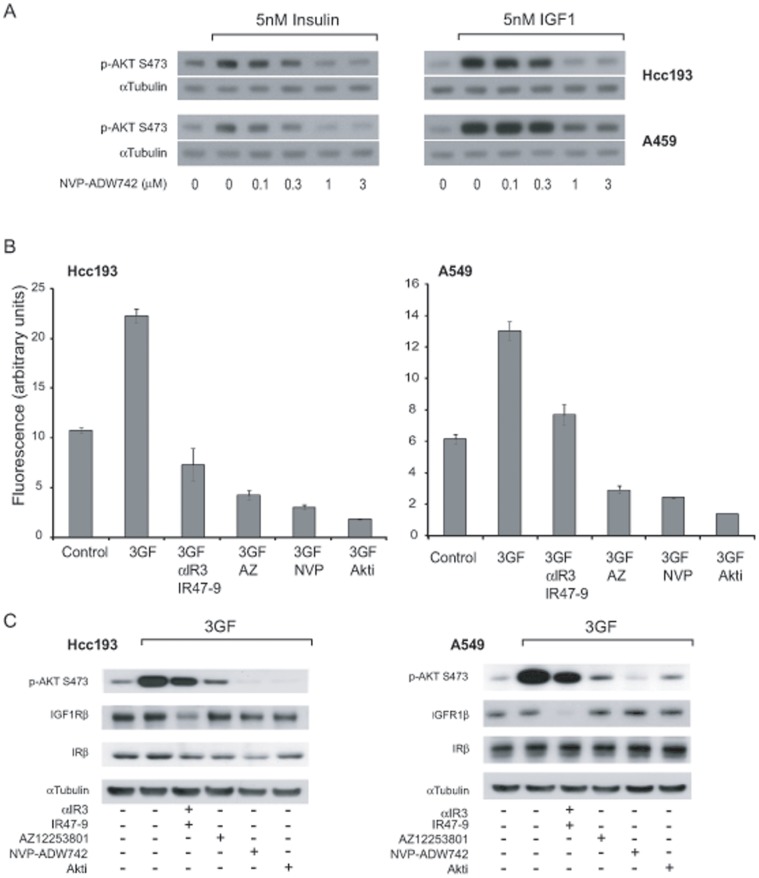
A second IGF1R/IR kinase inhibitor and an Akt inhibitor inhibit anchorage-independent proliferation more effectively than inhibition of the IGF1R/IR using monoclonal antibodies. (A) Hcc193 and A549 cells were treated with NVP-ADW742 (0.1 to 3 µM) for 2 h before 10 min incubation with 5 nM insulin or 5 nM IGF-1. Phosphorylation of Akt on S473 was determined by western blotting. An anti-α-tubulin antibody was used as a control for protein loading. (B) Hcc193 and A549 cells were cultured under anchorage–independent conditions in 0.1% serum in the presence of 3GF alone and in combination with αIR3 (2 µg/ml) and IR47-9 (2 µg/ml), AZ12253801 (50 nM), NVP-ADW742 (1 µM), or Akti-1/2 (10 µM) as indicated. After 5 days viable cells were estimated by the fluorescence of the metabolic reduction product of Alamar blue. Each bar represents the mean fluorescence of four replicate wells ± standard deviation from a single experiment. The results shown are representative of 2 independent experiments. ***, *p*<0.001; **, p<0.01 (valid for each experiment); two-tailed unpaired *t* test. (C) Hcc193 and A549 cells were treated with αIR3 (2 µg/ml) and IR47-9 (2 µg/ml) AZ12253801 (50 nM), NVP-ADW742 (1 µM) or Akti (10 µM) as indicated for 2 h before 10 min incubation with vehicle or 3GF. Phosphorylation of Akt on S473 and expression of IGFRβ and IRβ was determined by western blotting using specific antibodies. An anti-α-tubulin antibody was used as a control for protein loading.

In both Hcc193 and A549 cell lines AZ12253801 has a greater inhibitory effect on anchorage-independent proliferation than combined αIR3 and IR47-9. To investigate whether ATP-competitive inhibitors of IGF1R/IR provide a more effective mode of inhibiting growth-factor-induced cell proliferation than neutralising antibodies, the effect of a structurally distinct ATP-competitive small molecule IGF1R/IR inhibitor NVP-ADW742 on growth of Hcc193 and A549 was also assessed. NVP-ADW742 dose-dependently reduced 5 nM IGF-1 and 5 nM insulin-stimulated Akt phosphorylation in Hcc193 and A549 cells with a maximal effective concentration reached at 1 µM (Figure 4A).

Figure 4B illustrates the anchorage-independent proliferation of Hcc193 and A549 cells respectively in the presence of 3GF and the effect of αIR3 and IR47-9, AZ12253801 and NVP-ADW742 upon this. NVP-ADW742 inhibited 3GF-induced Hcc193 and A549 cell proliferation to a similar degree to AZ12253801 and to a greater degree than observed with a combination of αIR3 and IR47-9. This was reflected in the greater degree of inhibition of Akt phosphorylation by ATP-competitive inhibitors compared to αIR3/IR47-9 (Figure 4C).

The close correlation between the effect of AZ12253801, NVP-ADW742, αIR3 and IR47-9 on cell proliferation and on Akt phosphorylation suggested that Akt might contribute to 3GF-stimulated proliferation of the cells. To explore this further we used a selective allosteric inhibitor of Akt, Akti-1/2 on Hcc193 and A549 cells. Akti-1/2 was as effective as AZ12253801 and NVP-ADW742 at inhibiting anchorage-independent cell proliferation (Figure 4B) and similarly effective at inhibiting Akt phosphorylation (Figure 4C). This result is consistent with Akt mediating anchorage-independent proliferation of these cell lines downstream of the IGF1R and IR.

## Discussion

The IGF1-R has a well established role in tumorigenesis, however the recent failure of the IGF1-R monoclonal antibody figitumumab in NSCLC trials has questioned whether targeting this pathway alone will lead to a therapeutic benefit. Emerging evidence suggests that signalling through the related IR provides a mechanism by which tumour cells resist the effects of IGF1-R inhibition in human osteosarcoma and breast cancer. Consistent with this, our data support a potential role for the IR in proliferation of NSCLC cell lines with a high IR:IGF1R ratio. In addition we provide evidence that dual blockade of the IR and IGF-1R using an ATP-competitive small molecule IR/IGF-1R inhibitor has improved efficacy at inhibiting NSCLC cell proliferation over that obtained by simultaneous inhibition of the IR and IGF1-R using selective monoclonal antibodies.

Consistent with the established role of IGF1 in supporting tumour cell proliferation [17], [30], [31], [38], [48], neutralising antibody-directed blockade of the IGF1-R in each of the NSCLC cell lines tested resulted in a partial inhibition of proliferation (Figures 2–4). A549 cells were more sensitive than Hcc193 in this respect possibly reflecting the higher expression of IGF1R in A549 and the resultant low IR:IGF1R expression ratio. Selective blockade of the IR using a specific antibody also reduced proliferation in each cell line. In Hcc193 cells, which have a relatively high IR:IGF1R expression ratio, the extent of this reduction was similar to that caused by IGF1R inhibition, but in A549 cells the reduction in proliferation by blockade of the IR was markedly less than that seen upon IGF1R inhibition. These findings are consistent with a previous report showing that a breast cancer cell line with high IR:IGF1R expression ratio (MDAMB231) was more sensitive to IR blockade than a cell line with a low IR:IGF1R expression ratio (MCF7) [31]. Suppression of IR expression by RNA interference has also been shown to inhibit cell proliferation, the extent to which this occurs varying with cell type and nature of the assay [29], [30]. The IR therefore supports tumour cell proliferation of several cell types independently of the IGF1R, and we have here extended these observations to NSCLC cells.

Combined inhibition of the IGF1R and IR with monoclonal antibodies in the Hcc193 cell line reduced proliferation to a greater extent than inhibition of either receptor alone. This indicates that in NSCLC the IR has the potential to contribute to resistance to IGF1R inhibition by supporting cell proliferation. Such a role for the IR in the context of targeted IGF1R has been demonstrated in a transgenic mouse model of pancreatic neuroendocrine carcinogenesis and human breast cancer cells [31], and in osteosarcoma cell lines [30]. In both of these studies IGF2, rather than IGF1, was defined as the growth factor signalling through the IR to cause this protumorigenic effect. This may also be the case in NSCLC where IGF2 is known to support tumour growth [39], [49]. In addition, it is possible that insulin itself may contribute in this regard as it has well established mitogenic properties [23]–[26] and is included, with IGF1 and IGF2, in the growth medium in the current study. Blockade of the IR failed to enhance the effect of IGF1R inhibition in A549 cells suggesting that in NSCLC cases where low ratios of IR:IGF1R expression exist, and when those cells are exposed to IGF1 in addition to IGF2 and insulin, co-targeting the IR may not be beneficial.

Small molecule tyrosine kinase inhibitors such as AZ12253801, NVP-ADW742 and BMS754807 [50], [51] inhibit both the IGF1R and the IR and, as such, have the potential to be more effective anti-neoplastic agents than antibodies against IGF1R. Indeed, in the current study the effect of AZ12253801 on Hcc193 cells was consistent with that of dual IGF1R and IR blockade by antibodies as the extent of inhibition of proliferation was greater than seen upon blockade of the IGF1R alone. However, in both Hcc193 and A549 cell lines AZ12253801 was significantly more effective at inhibiting 3GF-stimulated Akt phosphorylation and cell proliferation than was the combination of αIR3 and IR47-9. A second, structurally distinct, small molecule IGF1R/IR inhibitor, NVP-ADW742 [52], was similarly effective as AZ12253801. Thus, direct kinase inhibition via ATP competition appears to provide improved efficacy in comparison to blocking ligand binding by antibody. Consistent with this, Dong et al demonstrated that targeting the IGF1R with a single inhibitory antibody (such as αIR3) had limited effect on Akt phosphorylation and cell proliferation induced through that receptor [53].

Nevertheless, we cannot exclude the possibility that each of AZ12253801 and NVP-ADW742 exhibit greater effects on proliferation than combined αIR3 and IR47-9 due to an off-target effect of the small molecule inhibitors on non-IR/IGF1R kinases. However, the fact that the individual effect of AZ12253801 *and* NVP-ADW742 on Akt phosphorylation is greater than with the combined antibodies suggests that this is unlikely to be the case. This is further supported by the Akt inhibitor, Akti1/2, being as effective as AZ12253801 and NVP-ADW742 at inhibiting 3GF-induced anchorage-independent cell proliferation. We and others have found Akt to be activated in NSCLC [54]–[56] and it is the subject of multiple drug discovery programs that have reached the clinical trial stage [57]. Our data when combined with similar observations in other cancer cell types [29], [31], [50], [53], [58] suggest that Akt is likely to have a principal role in mediating the proliferative effects of signalling from the IGF1R/IR. We cannot rule out, however, that the RAS/RAF/MAPK pathway also contributes to proliferation under these conditions.

Taken together, the results presented indicate that in NSCLC cells with a high IR:IGF1R ratio the IR contributes to driving tumour cell proliferation and that a small molecule IR/IGF1R inhibitor is a more effective approach to inhibiting cell proliferation. An alternative strategy to co-target the neoplastic functions of IGF1R and IR is ligand-targeting [5], [59]. Sequestration of IGF2 might be achieved through an IGF2-specific antibody [30], [60], or a soluble IGF2-specific ligand trap [61]. This approach avoids systemic inhibition of the IR thus enabling its function in energy and glucose homeostasis. However, it would be limited in tumours where cell proliferation or survival was supported by the local action of insulin itself.

A significant body of work predicts inhibition of the IGF1R to have anti-neoplastic effects yet such promise has not translated in to success in clinical trials, including that of the IGF1R antibody figitumumab in patients with advanced NSCLC [41], [62]. Advances in targeted therapy have highlighted the requirement to better understand the molecular basis of tumours and their subsets so that biomarkers predictive of clinical benefit can be identified. In NSCLC patients, Gualberto et al found higher pre-treatment levels of circulating free IGF1 to be predictive of the clinical benefit of figitumumab in chemotherapy [63]. Furthermore, cell line data [48] and preliminary clinical data from squamous tumours [64] indicate that higher expression of IGF1R may predict sensitivity to inhibitory antibodies against it. This latter point is consistent with the greater sensitivity of A549 cells than Hcc193 cells to αIR3 treatment observed in the current study. In addition, our study shows that the IR can support proliferation in NSCLC cell lines subject to IGF1R inhibition, and suggest that it is the ratio of IR:IGF1R expression that defines the sensitivity to inhibition of the IGF1R and indeed whether any benefit may be achieved from additionally targeting the IR. Furthermore, the finding that anti-IGF1R antibodies only partially inhibit tumour cell proliferation, whether added alone or in combination with anti-IR antibodies, suggests that approaches to inhibit ligand binding are suboptimal and may have contributed to the failure of figitumumab in clinical trials.

Our findings in NSCLC and similar evidence of a key role for the IR in other cancer types suggests that residual IGF1R and unencumbered IR signalling may have contributed to the lack of benefit of figitumumab in NSCLC trials. We propose therefore that a more effective approach to treat NSCLC will be through the use of small molecule ATP-competitive IR/IGF1R kinase inhibitors.
